# Impact of variants of uncertain significance on decision making about genetic testing for Hispanic males

**DOI:** 10.1016/j.gimo.2025.103480

**Published:** 2025-12-01

**Authors:** Jasmine Saunders, Veda N. Giri, Susan Vadaparampil, Adrian Rivera, Tatiana Sanchez Nolasco, Mariana Rangel Camacho, Nataliya Byrne, Kellie Owens, Michele Santacatterina, Stacy Loeb

**Affiliations:** 1 Department of Urology, New York University Langone Health, New York, NY; 2 Department of Internal Medicine, Section of Medical Oncology, Yale School of Medicine and Yale Cancer Center, New Haven, CT; 3 Department of Health Outcomes and Behavior, Moffitt Cancer Center, Tampa, FL; 4 Department Population Health, New York University Langone Health, New York, NY

**Keywords:** Genetic counseling, Genetic testing, Hispanic, Variants of uncertain significance

## Abstract

**Purpose:**

Underutilization of genetic testing among Hispanic males results in higher rates of variants of uncertain significance (VUS). We examined the impact of VUS on decision making and behavioral intentions.

**Methods:**

We conducted a nationwide survey of 807 US Hispanic males aged ≥40 in English and Spanish on perspectives about genetic testing results. Logistic regression was used to examine predictors of worry and behavior change with a hypothetical VUS result.

**Results:**

Over half of Hispanic male participants would still participate in genetic testing with a 1 in 5 chance of VUS. However, 36% were at least somewhat likely to regret testing and 49.9% would worry about cancer risk with VUS results. In addition, 56.3% were somewhat or very likely to change behavior due to a VUS, such as getting checked by the doctor more often or telling family members to get checked. Younger age and college education were associated with more worry and intended behavior change.

**Conclusion:**

Although many Hispanic males are interested in genetic testing despite the higher likelihood of VUS, potential consequences include decisional regret, anxiety, and even changes in behavior. Effective counseling and support are important for minoritized groups undergoing genetic evaluation to avoid the potential to exacerbate health disparities.

## Introduction

National data suggest that a significantly lower proportion of Hispanic/Latinx (hereafter referred to as Hispanic) adults undergo genetic testing compared with other ethnic groups.^1^ In addition, females are significantly more likely to undergo cancer genetic testing compared with males. This is primarily due to substantial underutilization of testing for Hereditary Breast and Ovarian Cancer genes in males,^1^ despite the importance of these genes for risk of multiple cancers (including breast, pancreatic, and prostate cancer).^2^

Prostate cancer is the most common cancer among Hispanic males in the US, comprising 22% of newly diagnosed cancers.^3^ Additionally, Hispanic males are more likely to be diagnosed at an advanced stage than non-Hispanic White males.^3^ The indications and importance of germline testing for prostate cancer have grown in recent years to include patients with high-risk or metastatic disease, Ashkenazi Jewish ancestry, or those who meet family history criteria, but there is underutilization among minoritized patient populations.^4^ One study found that only 2% of patients with prostate cancer who underwent germline testing were Hispanic.^4^ Genetic testing has the potential to affect screening or treatment protocols or prompt genetic testing of family members. However, the likelihood of inconclusive results, known as variants of uncertain significance (VUS), is significantly higher among Hispanic males (21.5%) compared with non-Hispanic White males (16.6%) undergoing germline testing for prostate cancer (*P* =.005).^5^

Given the high rates of VUS in Hispanic males undergoing genetic testing, it is important to understand the downstream impact of these results. Most studies of patient perspectives on VUS have focused on females with breast and ovarian cancer.^6^^,^^7^ Some prior studies have found that VUS results may have greater psychosocial impacts than other forms of genetic test results.^8^ Studies in females have also reported unnecessary prophylactic mastectomy in up to 50% with *BRCA1/2* VUS and average breast cancer risk,^9^ although they should not affect clinical management. Indeed, most VUS are ultimately reclassified as benign.^10^^,^^11^ However, there are limited data on the impact of VUS results for males.

Because of the higher likelihood of VUS findings and lack of data on downstream impact, the purpose of this study was to examine the perceptions around VUS among Hispanic males and the demographic factors associated with higher anxiety and behavioral changes in response to a VUS. We hypothesized that presentation of a hypothetical uncertain genetic test result would be associated with decisional regret, worry, and behavior change.

## Materials and Methods

We performed a cross-sectional survey of US Hispanic males following Institutional Review Board approval. Participants were recruited using Dynata, a nationwide online survey platform employed in prior studies.^12^ Eligibility criteria were US adult males age 18+ who identify as Hispanic. After obtaining informed consent, participants completed an electronic questionnaire in English or Spanish, via REDCap. Questions encompassed socio-demographics (eg, race, education level, and marital status), health literacy,^13^ and several other scales that are validated in both Spanish and English (eg, 5-item Familism Scale, Group-Based Medical Mistrust Scale).^13^, ^14^, ^15^ Additionally, the study team developed a 4-question survey examining perspectives on VUS, which was preceded by an introductory paragraph on what VUS is (see Table 1). We conducted forward- and back- translation in collaboration with a professional translation service.^16^ This was followed by a consensus process, including a review by a panel of bilingual experts and pilot testing with US Hispanic male adults (3 in English and 3 in Spanish), as in previous studies.^16^

**Table 1:** Perceptions of Hispanic males on variants of uncertain significance^a^

Survey Question	Very Unlikely (1)	Unlikely (2)	Neutral (3)	Somewhat Likely (4)	Very Likely (5)
How likely would you be to participate in genetic testing if there was a 1 in 5 chance that your results would be uncertain? (1)	56 (6.9%)	89 (11.0%)	211 (26.1%)	249 (31.0%)	202 (25.0%)
If you received an uncertain result, would you regret having taken the test? (2)	156 (19.3%)	180 (22.3%)	180 (22.3%)	189 (23.4%)	102 (12.6%)
If you received an uncertain result, would you feel worried about your risk of getting cancer (or getting cancer again if you have ever been diagnosed with cancer)? (3)	78 (9.7%)	102 (12.6%)	224 (27.8%)	257 (31.8%)	146 (18.1%)
If you received an uncertain result, would you change your behavior (for example, get checked by the doctor more often or tell family members to get checked)? (4)	57 (7.1%)	88 (10.9%)	207 (25.7%)	252 (31.2%)	203 (25.1%)

a The following introduction was provided for these questions: “Genetic tests have 3 main types of results: negative (not linked with cancer), positive (linked with cancer), and uncertain (we don’t know yet if your genes are linked to cancer without more research so do not have any recommendations for you at this time but may have updates in the future).” All questions used a 5 point Likert scale: 1 = very unlikely; 2 = unlikely; 3 = neutral; 4 = somewhat likely; 5 = very likely.

We used descriptive statistics to tabulate survey responses. Further analysis using Pearson’s chi-square test investigated associations between perceptions of VUS with baseline characteristics (English vs Spanish survey, age <50 vs 50+, White vs non-White race, US born vs not, married vs not, any insurance vs none, college graduate vs not, history of cancer vs none, and family cancer history vs none, as well as greater than or equal to the median vs less than median for health literacy, familism, and medical mistrust). In addition, multivariable logistic regression was performed to examine predictors of worry and changes in behavior with VUS. Responses of “somewhat likely” or “very likely” were grouped together as an indication of agreement.

## Results

807 Hispanic males completed the questionnaire, including 655 in English and 152 in Spanish. The mean age was 51.7, 65.3% of participants self-identified as White, 74.6% were born in the US, 58.7% were married, 89.3% had some form of insurance, 45.4% were college graduates or beyond, 10.8% had a personal history of cancer, and 46.5% had a family history of cancer.

Table 1 displays the VUS survey responses. With a 1 in 5 chance of uncertain results, 56% of respondents were “somewhat” or “very likely” to participate in genetic testing, with only 6.9% responding as “very unlikely.” However, 36% were at least “somewhat likely” to regret having done the test if they received a VUS result and 49.9% would feel worried about their risk of getting cancer. Additionally, 56.3% were “somewhat” or “very likely” to change their behavior due to a VUS, such as getting checked by the doctor more often or telling family members to get screening.

Chi-square analysis revealed that males under the age of 50 were more likely to feel worried about their risk of developing cancer after receiving an uncertain result (*P <*.01). Moreover, participants who were married (*P =*.04, *P <*.01), had a college degree (*P =*.01, *P <*.01), or who had a higher level of familism (*P <*.01, *P <*.01) were more likely to worry about their cancer risk and change their behavior in response to a VUS, respectively. Conversely, better health literacy scores were associated with less worry about developing cancer with a VUS (*P =*.03). On multivariable logistic regression (Table 2), age > 50 had significantly lower and college educated had significantly higher worry and intended behavior changed with VUS results. Higher familism was also associated with significantly greater worry about VUS.

**Table 2:** Multivariable logistic regression analysis for predictors of worry and behavior change in response to VUS for Hispanic males

Predictor	Worry OR (95% CI), *P* Value	Changes in Behavior OR (95% CI), *P* Value
(Intercept)	0.48 (0.25-0.92), *P =*.03	0.50 (0.26-0.96), *P =*.04
Age ≥ 50 years (ref: <50)	0.62 (0.46-0.84), *P* <.01^a^	1.42 (1.04-1.94), *P* =.03^a^
Married (ref: Not married)	1.11 (0.81-1.51), *P =*.53	1.27 (0.93-1.74), *P =*.14
College educated (ref: Not college educated)	1.42 (1.04-1.94), *P* =.03^a^	2.74 (2.05-3.68), *P* <.01^a^
Above-median Health literacy score	1.27 (0.94-1.71), *P =*.12	1.14 (0.83-1.58), *P =*.42
Above-median Familism score	2.77 (2.07-3.73), *P* <.01^a^	0.80 (0.58-1.09), *P =*.15
Above-median Medical Mistrust score	1.23 (0.90-1.68), *P =*.20	0.84 (0.62-1.14), *P =*.26
English language (ref: Spanish)	1.16 (0.78-1.73), *P =*.46	0.84 (0.57-1.25), *P =*.40
White race (ref: Non-White)	1.14 (0.83-1.56), *P =*.43	0.99 (0.72-1.35), *P =*.94
US born (ref: Foreign-born)	0.78 (0.54-1.11), *P =*.16	0.99 (0.69-1.41), *P =*.94
Health insurance (ref: No health insurance)	1.08 (0.67-1.76), *P =*.74	1.31 (0.81-2.13), *P =*.28
Personal history of cancer (ref: No history)	1.00 (0.61-1.63), *P* = 1.00	1.17 (0.71-1.94), *P =*.55
Family history of cancer (ref: No history)	1.03 (0.76-1.39), *P =*.86	1.06 (0.78-1.43), *P =*.72

a Indicates statistically significant results.

## Discussion

As uptake of genetic testing expands, it is important to understand how patients view VUS results. In our study of Hispanic males, we found that VUS may trigger regret, worry, and changes in health care behavior for many respondents, particularly those who are younger, college educated, and exhibit greater levels of familism. In a study by Culver et al,^17^ 3 months after genetic testing results disclosure (20% male, 37% Hispanic), patients with pathogenic variants had a more negative psychological response compared with VUS or negative results. Notably, VUS results were associated with significantly higher uncertainty scores compared with negative results. Although results were not presented separately for the subgroup with VUS, in the overall population older age and at least a college education were associated with lower Multidimensional Impact of Cancer Risk Assessment scores (ie, less impact). A previous study of females who underwent *BRCA1/2* testing found higher distress scores up to a year after receiving an uncertain result.^7^ A later study from Lumish et al^6^ investigated patients who received uninformative results from breast and ovarian cancer genetic panels; 3% of the participants were male and approximately 20% were Hispanic. They found that younger age, lower education level, and less genetic knowledge were associated with higher distress with a VUS.^6^ Our study also found that younger participants were more likely to worry about VUS results; however, lower education level was associated with decreased worry.

The literature and the results of our survey highlight the importance of thorough patient education and counseling about VUS to reduce unnecessary distress and inappropriate clinical mismanagement. An unexpected or unexplained VUS could lead to decisional regret, anxiety or even impact health-related behaviors, potentially exacerbating existing health care disparities. This is problematic particularly because many physicians lack understanding and education about the implications of VUS.^18^ Expanding public education about genetic evaluation is also important, including a need for more Spanish-language content in online platforms.^19^

A limitation of this study is the use of an online survey, excluding participation of individuals without internet access. Also, participants were asked about their hypothetical reaction to a VUS scenario, which could vary from patients’ actual response to a VUS with more extensive counseling, and the questions were not validated. Strengths include a large sample size of Hispanic males, who have a higher likelihood of VUS and yet are underrepresented in research. Additionally, offering the survey in English and Spanish enabled the evaluation of non-English preferring patients, who are less likely to receive genetic counseling^20^ and are therefore an important population to study. That notwithstanding, additional research is warranted into perspectives and impact of VUS in other ethnic groups, as well as addressing barriers to testing, optimal methods of reporting and tracking of VUS results, and effective modalities to provide education and support for patients and providers.

In conclusion, although many Hispanic males express willingness to participate in genetic testing despite the higher chance of a VUS, potential downstream consequences include decisional regret, anxiety, and changes in health behavior. Effective counseling and support are important for minoritized groups undergoing genetic evaluation to avoid the potential to exacerbate health disparities.

## Data Availability

Data are available for health/medical/biomedical research purposes. A formal letter of intent outlining research aims, rationale, approach, and documentation of local institutional review board approval should be submitted with the data request. A Data Use Agreement must be signed before any data are released.

## Conflict of Interest

Stacy Loeb reports consulting with Astellas, Blue Earth, Endo USA Inc, Savor Health and Doceree, and research funding from Endo USA Inc, unrelated to current study. Veda N. Giri reports grant funding from the Department of Defense and Prostate Cancer Foundation; stock ownership in Novopyxis (unrelated to this study). All other authors declare no conflicts of interest.
